# Comparing Handcrafted Radiomics Versus Latent Deep Learning Features of Admission Head CT for Hemorrhagic Stroke Outcome Prediction

**DOI:** 10.3390/biotech14040087

**Published:** 2025-11-02

**Authors:** Anh T. Tran, Junhao Wen, Gaby Abou Karam, Dorin Zeevi, Adnan I. Qureshi, Ajay Malhotra, Shahram Majidi, Niloufar Valizadeh, Santosh B. Murthy, Mert R. Sabuncu, David Roh, Guido J. Falcone, Kevin N. Sheth, Seyedmehdi Payabvash

**Affiliations:** 1 Department of Radiology, Columbia University Irving Medical Center, New York, NY 10032, USA; at4049@cumc.columbia.edu (A.T.T.);; 2 Department of Radiology and Biomedical Imaging, Yale School of Medicine, New Haven, CT 06520, USA; 3 Zeenat Qureshi Stroke Institute and Department of Neurology, University of Missouri, Columbia, MO 65212, USA; 4 Department of Neurosurgery, Icahn School of Medicine at Mount Sinai, New York, NY 10029, USA; 5 Department of Neurology, Weill Cornell Medical College, Cornell University, New York, NY 10065, USA; 6 Department of Radiology, Weill Cornell Medicine, New York, NY 10065, USA; 7 School of Electrical and Computer Engineering, Cornell Tech, Cornell University, New York, NY 10044, USA; 8 Department of Neurology, Columbia University Irving Medical Center, New York, NY 10032, USA; 9 Department of Neurology, Yale School of Medicine, New Haven, CT 06520, USA

**Keywords:** radiomics, latent deep features, U-net segmentation, generative auto-encoders, stroke, intracerebral hemorrhage

## Abstract

Handcrafted radiomics use predefined formulas to extract quantitative features from medical images, whereas deep neural networks learn de novo features through iterative training. We compared these approaches for predicting 3-month outcomes and hematoma expansion from admission non-contrast head CT in acute intracerebral hemorrhage (ICH). Training and cross-validation were performed using a multicenter trial cohort (n = 866), with external validation on a single-center dataset (n = 645). We trained multiscale U-shaped segmentation models for hematoma segmentation and extracted (i) radiomics from the segmented lesions and (ii) two latent deep feature sets—from the segmentation encoder and a generative autoencoder trained on dilated lesion patches. Features were reduced with unsupervised Non-Negative Matrix Factorization (NMF) to 128 per set and used—alone or in combination—for six machine-learning classifiers to predict 3-month clinical outcomes and (>3, >6, >9 mL) hematoma expansion thresholds. The addition of latent deep features to radiomics numerically increased model prediction performance for 3-month outcomes and hematoma expansion using Random Forest, XGBoost, Extra Trees, or Elastic Net classifiers; however, the improved accuracy only reached statistical significance in predicting >3 mL hematoma expansion. Clinically, these consistent but modest increases in prediction performance may improve risk stratification at the individual level. Nevertheless, the latent deep features show potential for extracting additional clinically relevant information from admission head CT for prognostication in hemorrhagic stroke.

## 1. Introduction

Radiomics refers to the extraction of handcrafted quantitative features from medical images using predefined formulas representing lesion shape, intensity, and texture [1,2,3]. In contrast, deep learning models such as convolutional neural networks (CNNs) automatically learn hierarchical representations from clinical scans through iterative training, without any preset formula [4,5]. Compared to radiomics, deep learning models can capture high-order abstract patterns or subtle context in images [6,7,8]. However, training of CNN models requires large sample sizes, which can be particularly challenging in less common medical conditions. To circumvent this, some groups have used pretrained CNNs to extract features from clinical scans [9,10,11]. This strategy is—at least theoretically—limited by task specificity and by the fact that most pretrained CNNs are optimized for 2D non-medical images, making direct application to 3D volumetric clinical scans imperfect. Alternatively, a U-shaped neural network for a segmentation task or configured as an autoencoder can extract latent deep features from medical images [12,13,14]. Compared with generic pretrained CNNs, such in-domain models provide modality- and disease-specific features that can support clinical prediction.

Latent deep features extracted from brain MRIs have been widely used for the classification and differentiation of cerebral tumors. Various U-Net–based segmentation models have been applied for the dual purpose of lesion segmentation and prediction of pathological grades or tumor subtypes [15,16,17,18]. Denes-Fazakas et al. developed L-net, which couples a U-Net segmentation unit with a CNN classification unit so that the CNN uses latent deep features extracted by the U-Net rather than the raw image [15]. Rai et al. proposed a “two-headed” UNet-EfficientNet architecture that performs segmentation and classification in parallel, leveraging latent deep feature extraction from the segmentation arm for the classification of brain tumors [16]. In addition, several groups have utilized generative autoencoders to capture texture-based features for brain tumor classification [19,20,21,22,23,24,25,26,27]. For example, Cheng et al. reported on a multimodal disentangled variational autoencoder for glioma grading [19]. Ullah et al. combined CNN architecture with a Stack Encoder–Decoder network to extract and apply multimodal brain MRI features for tumor classification [22]. However, this approach has rarely been extended beyond oncologic neuroimaging. In this study, we apply such strategies and compare them with handcrafted radiomics for outcome prediction in hemorrhagic stroke.

Spontaneous acute intracerebral hemorrhage (ICH) accounts for 49.5% of the stroke burden in terms of disability-adjusted life years [DALYs] loss and 45.6% of stroke-related mortality [28]. Hematoma expansion affects up to 30% of ICH patients within the first 24 h after onset and is a main modifiable risk factor and treatment target in ICH [29]. Prior studies have shown that radiomic features extracted from admission head CT can predict hematoma expansion, long-term outcomes, and mortality in ICH [30,31,32,33,34,35,36,37,38,39,40,41]. For this study, we extracted the handcrafted radiomic features from the hematoma lesions on admission head CTs for the prediction of 3-month clinical outcome and >3-, 6-, and 9 mL hematoma expansion. We compared these radiomic features with latent representations derived from (i) a U-Net–based ICH segmentation network [42] and (ii) a generative adversarial autoencoder trained to reconstruct the hemorrhagic lesion and perihematomal brain tissue [43]. U-Net segmentation models learn hierarchical representative features distinguishing hemorrhage from normal brain, where the encoder concentrates salient information and the decoder produces voxel-wise labels [44,45]. Similarly, U-shaped generative autoencoders reconstruct the input by learning a compact latent representation of cropped head CT patches containing the hemorrhage and perihematomal tissue, thereby capturing lesion morphology and surrounding tissue context [46,47]. We applied the unsupervised Non-negative Matrix Factorization (NMF) feature extraction to both radiomics and latent deep features [48]. Notably, in our study design, both feature extraction—via radiomics, U-Net segmentation, or generative autoencoder—and feature selection are performed independent of outcome prediction (i.e., 3-month clinical outcome or hematoma expansion), thereby avoiding information leakage and yielding outcome-agnostic features that can be reused for other ICH endpoints. We compared these features as input for six different machine learning classifiers.

## 2. Materials and Methods

### 2.1. Patients’ Datasets

For training and cross-validation, we used the patients’ dataset from the Antihypertensive Treatment of Acute Cerebral Hemorrhage II (ATACH-2) clinical trial [49]. ATACH-2 was a large-scale, randomized, multicenter study designed to assess the clinical impact of intensive systolic blood pressure lowering in individuals with acute ICH but found no treatment benefits [49]. For independent validation, we used the Yale Longitudinal Study of Acute Brain Injury dataset [50]. We included adult patients (>18 years old) with acute spontaneous ICH and baseline and follow-up head CT scans. For follow-up CT, we used the scan taken close to 24 h after the ICH onset. Subjects with missing follow-up scans or poor-quality CTs were excluded. The 3-month modified Rankin Scale (mRS), or the closest available follow-up, was used to determine clinical outcome. Poor outcome was defined as mRS > 3, consistent with the prior literature [49].

### 2.2. The U-Shaped Hematoma Segmentation Model

We used nnU-Net [42] for the automated segmentation of hematomas on baseline non-contrast head CTs. The nnU-Net is a self-adapting deep learning framework that automatically configures preprocessing, network architecture, and training settings based on the provided dataset. The pipeline of nnU-Net consists of the following key components:

- Preprocessing: Standardized intensity normalization, resampling to isotropic voxel spacing, and automatic cropping based on region of interest.
- Network Architecture: A fully convolutional encoder–decoder model with residual blocks and deep supervision for improved gradient flow.
- Training Strategy: Dice loss and cross-entropy loss are combined to address class imbalance, ensuring accurate segmentation of small hematomas.

**Figure 1:**
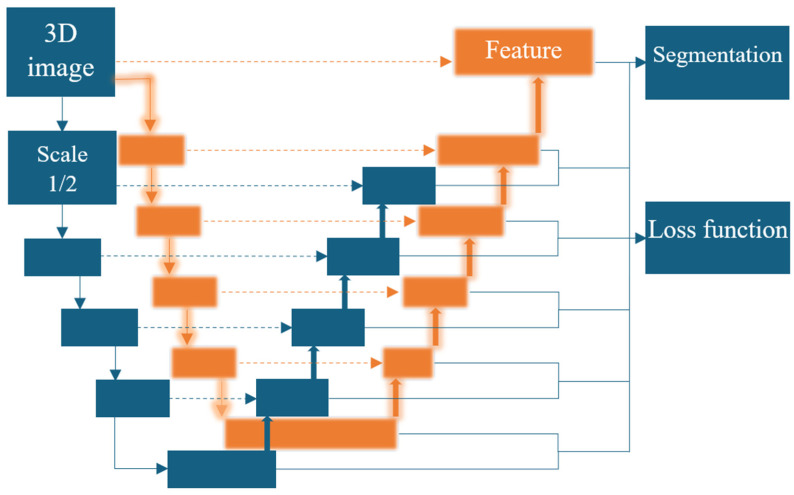
Integrating a multiscale model into nnU-Net with multiple outputs for loss function. The final nn-UNET model included both the original and the ½ scale head CT images for segmentation of hematomas.

The ground truth hematoma masks for training and validation were based on manually delineated hemorrhagic lesions. We adopted a multiscale nnU-Net architecture (Figure 1) for ICH segmentation to improve the network’s capacity to capture a broader and more diverse range of features across multiple spatial resolutions [51]. For the multiscale approach, we preprocessed input head CT scans at two isotropic spaces: 128 × 128 × 128 and 64 × 64 × 64. Each scale passes through an encoder to extract rich hierarchical features, with latent features of (512, 8, 8, 8) and (512, 4, 4, 4), respectively. By processing both the original and scaled versions of the input images through parallel encoder paths, the network learns to capture high-level contextual features from the down-sampled image while simultaneously preserving fine spatial details from the original resolution. This dual-path encoding strategy allows the final layers of the two encoders to extract features derived from full-resolution and scaled representation. Incorporating multiscale inputs theoretically allows the network to more accurately differentiate small hematomas from adjacent brain tissue, thus improving segmentation performance. This capability can be crucial for ICH segmentation, where lesions can vary widely in size, shape, and anatomical location. We assessed the model’s segmentation performance using the Dice Similarity Coefficient, which measured the overlap between the model’s segmentation predictions and manually delineated ground truth hematoma masks [52].

### 2.3. Extraction of Handcrafted Radiomic Features

Radiomic features are quantitative descriptors extracted from medical images that capture various properties of a target lesion, including its shape, intensity distribution, texture, and spatial heterogeneity [53]. We applied the pyradiomics package [54] to extract n = 1693 radiomic features from hematoma lesions on admission non-contrast head CT scans (detailed list in Supplemental Table S1). These features quantified the following characteristics of hematomas:

- Shape-based Features: Quantifying the geometry of the region of interest (ROI), such as volume, surface area, and sphericity.
- First-order Statistics (Intensity-based): Quantifying the distribution of voxel intensities within the ROI, such as mean, median, variance, skewness, kurtosis, entropy, and energy.
- Texture Features (second-order and higher): Capturing spatial relationships between lesion voxels, such as Gray-Level Co-occurrence Matrix (GLCM), and Gray-Level Run Length Matrix (GLRLM).
- Wavelet/Filter-based Features: Applying transforms such as wavelet or Laplacian of Gaussian to reveal multiscale features.

### 2.4. Extraction of Latent Deep Learning Features from nnU-Net

**Figure 2:**
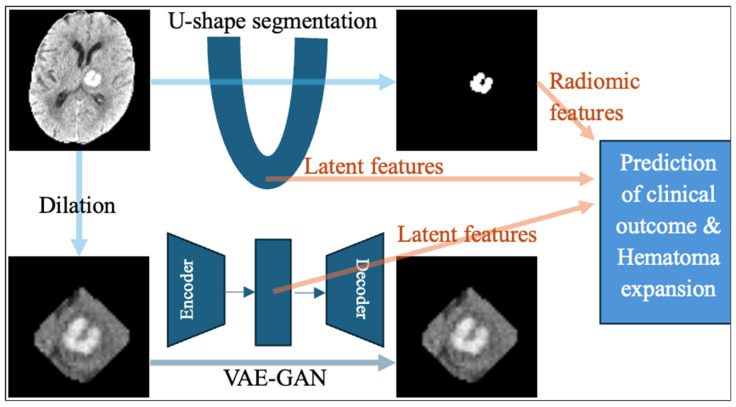
The overall pipeline for extraction of radiomic features and latent deep learning features. The radiomic features were extracted from the hematoma lesion segmented by nnU-Net. A set of latent deep learning features were extracted from the encoder of the U-shaped segmentation neural network at the bottleneck. Then, dilated masks of the hematoma were used as input for a Variational Autoencoder–Generative Adversarial Network (VAE-GAN), and an additional set of latent deep features were extracted from the encoder bottleneck as the model regenerates the CT images within the dilated hematoma mask.

We extracted high-level features from the final layer of the encoder in the nnU-Net segmentation model (Figure 2). These features tend to capture the most abstract semantic representations of the input image. At this stage of the U-shaped network, the spatial dimensions of the feature map have been significantly down-sampled through pooling or stride convolution operations, resulting in lower spatial resolution but much richer contextual and semantic understanding. These deep features encode global information about the entire image, such as object presence, class identity, and relationships between classes, rather than local textures or edges. In the context of ICH segmentation, these features can capture the overall shape, distribution, and anatomical context of hemorrhagic regions. This is especially important for distinguishing ICH from other tissues or pathologies that may appear similar at the local scale. In the U-shaped model, these features are typically passed through a bottleneck before being fed into the decoder arm, which will reconstruct the segmentation map by gradually increasing the spatial resolution through sampling and convolution.

### 2.5. Extraction of Latent Deep Features from a Generative Adversarial Network Autoencoder

Latent features from the U-shaped segmentation network theoretically capture characteristics that distinguish hematoma from surrounding brain parenchyma. To extract additional texture information, we applied an autoencoder reconstruction model using cropped CT images capturing hematoma and surrounding tissue with a dilated mask (Figure 2). Dilating the ICH region allows the model to capture surrounding tissue changes or edema, which may be clinically relevant. Specifically, we apply binary dilation with a structuring element size 5 to the predicted ICH mask, expanding the lesion region to capture surrounding contextual features. This dilated mask is then used to crop and extract an ROI from the original head CT at 64 × 64 × 64 size. This enriched region is then passed through a Variational Autoencoder–Generative Adversarial Network (VAE-GAN) architecture [43] to compress and model its latent features (Figure 2). The VAE-GAN combines the strengths of VAEs (robust probabilistic latent space encoding) with those of GANs (high-quality image generation) to create compact, expressive feature representations that are both informative and generative.

### 2.6. Unsupervised Feature Selection

Unsupervised feature selection can identify a subset of relevant features from high-dimensional data without relying on labeled information [55]. This approach is essential where labeled data is scarce or unavailable, such as for clustering, anomaly detection, and exploratory data analysis. Unlike supervised techniques that use class labels to guide feature selection, unsupervised methods leverage data properties such as variance, correlation, or latent structure. The most notable technique used in this context is Non-negative Matrix Factorization (NMF) [48]. NFM can simplify complex data by breaking it down into smaller parts, while ensuring all values remain non-negative. NMF helps highlight important patterns by grouping features that often appear together, making it useful for understanding the structure of the data and selecting key features without supervision. In this study, we applied NMF to reduce the high-dimensional latent features extracted from (1) radiomic features; (2) two different scales of the nnU-Net segmentation model—compressing features of 512 × 8 × 8 × 8 and 512 × 4 × 4 × 4 size; and (3) autoencoder latent features of 512 × 4 × 4 × 4 size, into 128 dimensions each. The three 128-dimensional feature sets were used separately or combined into a single feature vector as inputs for the final machine learning models.

### 2.7. Machine Learning Prediction Models

Given the diversity of real-world data—ranging from linear to highly nonlinear relationships—it is beneficial to evaluate a variety of models with different underlying assumptions and learning strategies. We implemented six diverse models: Random Forest (RF) with 1000 estimators [56], XGBoost with 1000 estimators and log-loss as the evaluation metric [57], Naive Bayes classifier (GaussianNB) [58], Extra Trees Classifier (ExtraTrees) with 1000 estimators [59], Elastic Net regularization (ElasticNet_LogReg) with a balanced mix of L1 and L2 penalties (l1_ratio = 0.5) and the “saga” solver [60], and Support Vector Machine (SVM) with a radial basis function (RBF) kernel [61]. All models were initialized with default settings and a fixed random seed to ensure reproducibility, enabling a fair comparison across classifiers. We applied a stratified 5-fold cross-validation to obtain the hyperparameters using the ATACH-2 dataset. Then, we applied the hyperparameters to the entire ATACH-2 dataset for final training and subsequently applied the final model to the independent Yale test set. In each step of cross-validation and final testing, NMF dimensionality reduction was applied to the training fold or training set and then tested on the validation fold or independent test cohort. Due to outcome imbalance, with substantially fewer patients experiencing poor outcomes or hematoma expansion, we implemented stratified 5-fold cross-validation on the ATACH-2 cohort based on the corresponding outcome labels. Stratification ensures that each fold preserves approximately the same proportion of positive and negative cases, preventing training folds from being dominated by the majority class.

We trained and validated these machine learning classifiers using seven different inputs: (1) all radiomic features, (2) only shape radiomic features, (3) latent features from nnU-Net, (4) latent features from VAE-GAN, (5) combination of latent features from both nnU-Net and VAE-GAN, (6) combination of radiomic and latent features from nnU-Net and VAE-GAN, and (7) combination of radiomic shape and latent features from nnU-Net and VAE-GAN. The models were trained to predict four different binary outcomes: (a) 3-month poor outcomes, and (b) >3 mL, (c) >6 mL, and (d) >9 mL hematoma expansion. Model performances were quantified and compared using the receiver operating characteristics (ROCs) area under the curve (AUC) [62].

### 2.8. Statistical Analysis

Continuous variables are reported as mean ± standard variation and categorical variables are reported as number (percentage). Additional statistical analyses were performed to verify whether observed differences in classifier performance across feature pipelines were statistically significant [63,64]. Because model performance metrics (e.g., AUC) across repeated cross-validation folds and classifiers did not meet normality assumptions (Shapiro–Wilk test, *p* < 0.05), we used non-parametric tests. A Friedman test was first applied to assess global differences among the seven feature-extraction pipelines across the six classifiers for each outcome label (3-month mRS and hematoma expansion thresholds of 3 mL, 6 mL, and 9 mL) [65]. When the Friedman test indicated a significant global effect (*p* < 0.05), pairwise Wilcoxon signed-rank tests were performed between pipelines to identify the source of the difference. To control for multiple comparisons, *p*-values were adjusted using three complementary procedures: Bonferroni, Holm–Bonferroni, and Benjamini–Hochberg (False Discovery Rate, FDR) [66].

## 3. Results

### 3.1. Patients’ Characteristics

A total of 866 patients from the ATACH-2 trial were included in the training/cross-validation cohort and 645 from the Yale dataset in the independent test cohort. Patients’ characteristics are summarized and compared between the two cohorts in Table 1. Overall, patients in ATACH-2 had smaller initial hematoma volume, less severe symptoms at presentation, and lower rates of hematoma expansions and poor outcomes. This is likely due to trial inclusion criteria limiting enrollment to those with <60 mL ICH at admission.

### 3.2. Automated Hematoma Segmentation Performance

We used baseline head CTs for training and validation. The multiscale nnU-Net using dual input resolutions achieved an average Dice of 0.88 ± 0.05 in cross-validation and 0.77 ± 0.19 in independent tests. Notably, a single scale nnU-Net also achieved an average Dice of 0.87 ± 0.08 in cross-validation and 0.77 ± 0.19 in independent tests.

### 3.3. Comparison of Radiomics and Latent Deep Features in ICH Outcome Prediction

**Figure 3:**
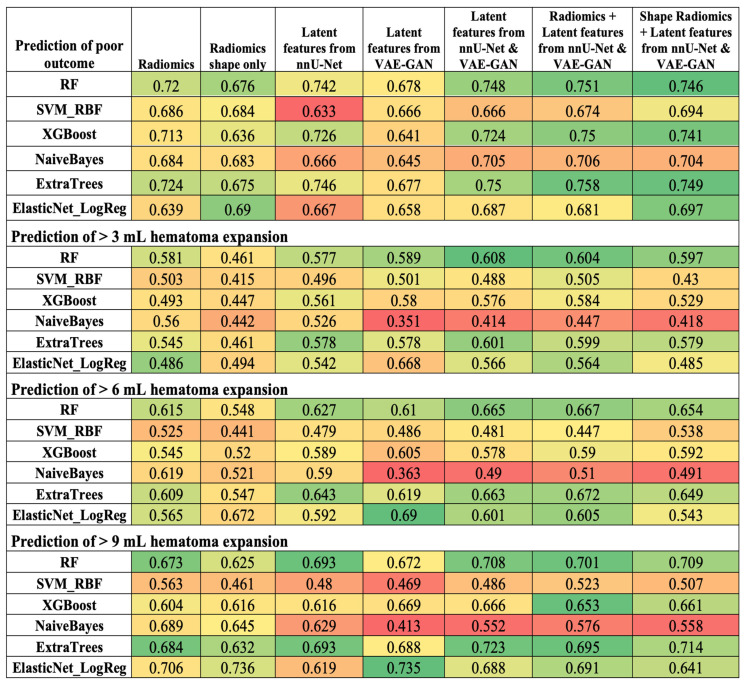
The AUCs of each prediction model with different inputs in independent tests for functional outcome and >3-, >6-, and >9 mL hematoma expansion. Green indicates higher performance, yellow indicates moderate performance, and red indicates lower performance values. Darker green corresponds to the best-performing models within each prediction task.

Figure 3 summarizes the accuracy of machine learning models with different inputs in the prediction of outcome and hematoma expansion. Supplementary Table S2 includes the details of AUC, F1-score, sensitivity, specificity, and negative and positive predictive values for all iterations among both validation folds and the independent test cohort.

Overall, combined latent deep features extracted by the nnU-Net and VAE-GAN consistently but only slightly had higher AUCs than radiomics in Random Forest, XGBoost, Extra Trees, and logistic regression. Supplementary Table S3 shows the detailed AUC differences between radiomics alone versus a combination of radiomics with latent deep features as input for all classifiers. The XGBoost and Elastic Net models using combined input had significantly higher AUCs in predicting >3 mL hematoma expansion (*p* = 0.027 and 0.040, respectively). Otherwise, the higher AUCs did not reach statistical significance. Notably, the Navie Bayes models in the prediction of hematoma expansion had lower AUCs using combined inputs compared to radiomics alone.

The Friedman test showed a statistically significant global difference in classifier AUCs between pipelines in predicting 3-month outcome with X^2^ = 20.429, *p* = 0.002. No significant global difference was observed in classifier AUCs between pipelines in predicting >3 mL (X^2^ = 10.286, *p* = 0.113), >6 mL (X^2^ = 4.143, *p* = 0.6573), or >9 mL (X^2^ = 3.786, *p* = 0.706) hematoma expansion. As detailed in Supplementary Table S5, we then compared the seven pipelines across the six classifiers using a signed-rank pairwise Wilcoxon test with Bonferroni correction, Holm–Bonferroni correction, and Benjamini–Hochberg by controlling the False Discovery Rate (FDR). Although none of the comparisons reached statistical significance after correction, pipelines with latent features showed consistent numerical improvement over the baseline. These findings suggest the potential for improved performance by combining latent features with radiometric geometric features and shape descriptors, although further validation with larger sample sizes and more classifiers is needed.

**Figure 4:**
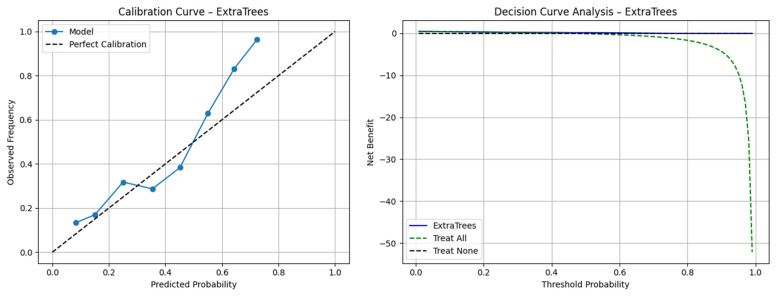
The example calibration curves and decision curve of the best model for poor outcome prediction (Extra Trees model with radiomics and latent deep features from nnU-Net and VAE-GAN).

**Figure 5:**
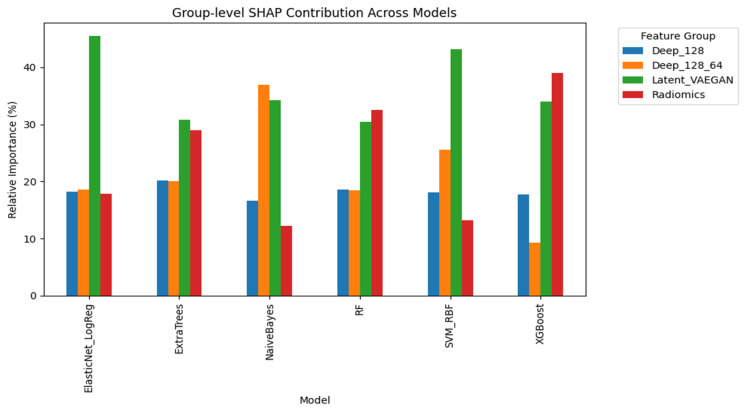
The group-level SHAP contribution across models in outcome prediction.

The calibration curve analysis for the best-performing model, the Extra Trees classifier trained with a combination of radiomic and latent deep features from both nnU-Net and VAE-GAN encoders, demonstrated great similarity between the predicted and observed probabilities of poor outcome. The calibration plot showed close alignment across the entire probability range, with minimal overestimation in the highest risk decile, indicating that the model’s predicted risk closely reflected the true event rate (Figure 4). We also compared the models’ performance between male versus female patients and found no significant difference (Supplementary Table S4). Model interpretability using SHAP (Shapley Additive Interpretation) revealed consistent feature group importance patterns across all classifiers in the outcome prediction task. In addition to the importance of radiomic features, the latent deep features obtained from the VAE-GAN encoder contributed the most in terms of predictive importance, capturing subtle contextual and perivascular texture patterns beyond handcrafted radiomic features (Figure 5).

## 4. Discussion

Our proposed models for predicting hematoma expansion and clinical outcomes from admission head CT scans can risk-stratify patients and potentially guide early treatment or intervention immediately after ICH diagnosis, even in the absence of additional clinical information. We found that adding latent deep features to radiomics slightly increased model accuracy, though not to a statistically significant degree. Nevertheless, SHAP explainability analysis indicated that, when combined, latent deep features exert similar or even greater impact on prediction decisions compared with radiomics. Overall, our findings demonstrate promising but not definitive added value of latent deep features for prognostic modeling in hemorrhagic stroke. Furthermore, because the latent features are outcome-independent and do not rely on follow-up data, this framework can be repurposed as a prognostic tool to predict future hematoma expansion or other downstream outcomes.

We trained a multiscale nnU-Net for hematoma segmentation on non-contrast head CT. We showed that dual-scale inputs improved Dice accuracy over single-scale, and latent features from the dual-scale nnU-Net likewise improved prediction performance. Similar multiscale input fusion [67] and multiscale densely connected U-Net [68] architecture have previously been shown to improve segmentation performance. To design a fully automated pipeline, nnU-Net outputs were used to extract both radiomics and latent deep features. Encoder features from segmentation nnU-Net capture hemorrhage morphology and its distinction from perihematomal tissue, while a generative autoencoder (VAE-GAN) trained on dilated lesion patches learns latent vectors encoding spatial texture and context from ICH and surrounding brain tissue. Concatenating these vectors yields a set of composite latent deep feature that can substitute—or augment—conventional radiomics. The addition of the 14 shape features provided almost similar incremental gain in prediction accuracy to the full set of radiomics. Using unsupervised NMF, we reduced the feature space to 128 variables, which is practical for studies with limited sample sizes. Overall, we demonstrate the feasibility of capturing prognostically relevant shape and texture information in <300 (latent deep) features that can be flexibly applied to any ICH outcome prediction scenario.

Prior studies have shown that radiomics from admission non-contrast head CT and deep learning models can predict hematoma expansion [69,70,71]. In head-to-head comparisons, deep learning models outperform radiomics alone [37]. Similarly, radiomics-based and deep learning models can predict clinical outcome from admission head CT scans [32]. Complementary evidence suggests machine learning models that incorporate clinical data further improve prediction [32]. Although U-Net-based segmentation and generative autoencoders have been used in brain tumor imaging to create composite latent deep features [15,16,17,18,19,20,21,22,23,24,25,26,27], this strategy has barely been applied to ICH. Lee et al. applied a combination of ICH detection and segmentation deep features to predict hematoma expansion in 572 patients [72]. We also introduce unsupervised NMF for feature selection and systematically compare radiomics and latent deep features, both separately and in combination. Our findings demonstrate the feasibility of an outcome-agnostic latent deep feature extraction/selection pipeline that yields a compact feature set that is suited to small-sample ICH studies.

While the combination of radiomics and latent deep features from nnU-Net segmentation and a VAE-GAN autoencoder consistently achieved numerically higher AUCs in Random Forest, XGBoost, Extra Trees, and Elastic Net models compared to radiomics alone, the absolute gains were statistically significant only in the prediction of >3 mL hematoma expansion. Clinically, these differences may improve risk stratification at the individual level; nevertheless, the latent features show potential for extracting additional prognostic information from medical images, given their impact on model prediction decision in SHAP analysis. In addition, the combination of latent deep features from segmentation and the autoencoder showed a trend towards higher accuracy than radiomics, suggesting that they may potentially be substitutes for handcrafted features.

Notably, despite significant demographic, clinical, and radiological differences between the ATACH-2 and Yale dataset, we found no consistent decline in model performance when comparing the validation and independent test cohorts (Supplementary Table S3). Compared with the ATACH-2, the Yale group was significantly older, had a higher proportion of Hispanic and white patients, and had higher hypertension, diabetes, hyperlipidemia, and atrial fibrillation. Patients in the Yale dataset had more severe neurological symptoms, reflected by lower Glasgow Coma Scale and higher NIH Stroke Scale scores. Hematoma volumes were also larger in the Yale group, with larger initial and follow-up volumes, and hematoma expansion rates were higher at all thresholds (>3, >6, and >9 mL). These differences represent a clear distributional shift between the training (ATACH-2) and validation (Yale) domains. While this supports the generalizability of the models, it also shows that the slight shift in calibration may impact the transferability of learned image representations, particularly for latent deep features that are more sensitive to intensity patterns. However, our models retained predictive performance between cross-validation and the independent test set.

The latent features from the U-shaped model tend to capture characteristics that differentiate the hematoma from the surrounding parenchyma, whereas the VAE-GAN model using the dilated lesion masks for input encodes both the texture within and around the hematoma, as well as broader contextual changes. This enables the combined features to incorporate information related to edema, boundary irregularities, and tissue heterogeneity. However, these features are not directly interpretable by humans. To enhance transparency, saliency-based visualization methods (e.g., 3D Grad-CAM) can be applied to the expanded lesion to identify the brain areas most influential in the latent features.

A main limitation of our study was clinical differences between the training/cross-validation cohort from ATACH-2 and the independent test set from Yale, as shown in Table 1. However, the heterogeneity of the multicentric training cohort with a fully independent test set supports the generalizability of our final models. Given that our models were intentionally image-only, we did not include clinical variables; prior work (including ours [32]) has shown that the combination of imaging and clinical variables improves prediction accuracy. It should also be noted that latent deep features—especially after NMF selection—are less interpretable than radiomics.

## 5. Conclusions

Using a large multicenter cohort for training and cross-validation, with an independent test set, we demonstrated that adding latent deep features from a segmentation encoder and a generative autoencoder to handcrafted radiomics yielded slightly more accurate predictions of outcomes and hematoma expansion, although statistical significance was reached in only a few instances. When combined features were used as input, latent deep features contributed equally or more to the machine-learning predictions. Our proposed pipeline—comprising outcome-agnostic feature extraction, unsupervised NMF dimensionality reduction, and automated segmentation—can be readily deployed in ICH studies with limited sample sizes.

## Figures and Tables

**Table 1:** Summary of patients’ characteristics in (ATACH-2) training/cross-validation and (Yale) independent test cohorts.

		ATACH-2 (n = 866)	Yale (n = 645)	*p* Values
3-month poor outcome		316 (36.5%)	301 (46.7%)	<0.001
>3 mL hematoma expansion		98 (11.3%)	163 (25.3%)	<0.001
>6 mL hematoma expansion		79 (9.1%)	122 (18.9%)	<0.001
>9 mL hematoma expansion		53 (6.1%)	97 (15.0%)	<0.001
Sex [male]		528 (60.9%)	354 (54.9%)	0.020
Age [years]		62.1 ± 12.9	69.6 ± 14.4	<0.001
Hispanic		69 (8.0%)	329 (52.2%)	<0.001
Race	White	241	440	<0.001
	Black	110	125	
	Asian	489	17	
	Other	26	63	
Systolic blood pressure [mmHg]		175.2 ± 25.1	172.9 ± 32.9	0.147
History of hypertension		690 (79.7%)	548 (85.0%)	0.010
History of diabetes mellitus		166 (19.2%)	173 (26.8%)	<0.001
History of hyperlipidemia		213 (24.6%)	346 (53.6%)	<0.001
History of atrial fibrillation		29 (3.4%)	139 (21.6%)	<0.001
Baseline Glasgow Coma Scale	3–11	127 (14.7%)	179 (27.8%)	<0.001
	12–14	242 (27.9%)	168 (26.1%)	
	15	497 (57.4%)	270 (41.9%)	
	unknown		28 (4.3%)	
Baseline NIH Stroke Scale score	0–4	137 (15.8%)	181 (28.1%)	<0.001
	5–9	226 (26.1%)	110 (17.1%)	
	10–14	235 (27.1%)	76 (11.8%)	
	15–19	159 (18.4%)	86 (13.3%)	
	20–25	77 (8.9%)	67 (10.4%)	
	>25	27 (3.1%)	38 (5.9%)	
	unknown	5 (0.6%)	87 (13.5%)	
Baseline hematoma volume [mL]		13.1 ± 12.6	18.7 ± 20.7	<0.001
Follow-up hematoma volume [mL]		15.8 ± 16.7	23.0 ± 25.9	<0.001
CT scans	Slice thickness [mm]	5.3 ± 1.8	4.8 ± 0.7	<0.001
	Min axial image matrix [n x n]	[418 × 418]	[472 × 472]	
	Max axial matrix [n x n]	[512 × 734]	[1024 × 1024]	
	Number of slices	31.0 ± 18.0	35.1 ± 11.5	<0.001

Continuous variables are reported as mean ± standard variation and compared between the two cohorts using the *t* test; categorical variables are reported as number (percentage) and compared be-tween the two cohorts using the Chi square test.

## Data Availability

The original contributions presented in this study are included in the article/Supplementary Materials. Further inquiries can be directed to the corresponding author(s).

## Supplementary Materials

The following supporting information can be downloaded at https://www.mdpi.com/article/10.3390/biotech14040087/s1, Supplementary Table S1: List of radiomic features; Supplementary Table S2: Details of machine learning classifier performance; Supplementary Table S3: Comparison of models with radiomics alone versus those with combined radiomics and latent deep features; Supplementary Table S4: Comparison of model performance between male versus female patients; Supplementary Table S5: Statistical comparison of different models.

## Abbreviations

The following abbreviations are used in this manuscript:

ATACH-2	Antihypertensive Treatment of Acute Cerebral Hemorrhage II
AUC	Area under the curve
CNN	Convolutional Neural Networks
DALYs	Disability-adjusted life years
FDR	False Discovery Rate
GLRLM	Gray-Level Run Length Matrix
GLCM	Gray-Level Co-occurrence Matrix
ICH	Intracerebral hemorrhage
NMF	Non-negative Matrix Factorization
RBF	Radial basis function
ROC	Receiver operating characteristics
ROI	Region of interest
RF	Random Forest
SVM	Support Vector Machine
VAE-GAN	Variational Autoencoder–Generative Adversarial Network
SHAP	Shapley Additive Interpretation
