# Supplementary material for: Comparing Handcrafted Radiomics Versus Latent Deep Learning Features of Admission Head CT for Hemorrhagic Stroke Outcome Prediction
Source: BioTech (Basel). 2025 Nov 2;14(4):87. doi: 10.3390/biotech14040087 (PMC12641684; doi:10.3390/biotech14040087)
Supplement: Supplementary file 1 [file biotech-14-00087-s001.zip › Supplementary Table 1.pdf]

Supplemental Table S1. List of radiomics features extracted from hematoma on admission non-contrast head CT (Total=1693)

Group	Number of features	Group Description
original_glcm	24	Gray-Level Co-occurrence Matrix (GLCM) extracted from Original Image
exponential_glcm	24	Gray-Level Co-occurrence Matrix (GLCM) extracted from exponential
lbp-3D-k_glcm	24	Gray-Level Co-occurrence Matrix (GLCM) extracted from 3D Local Binary Pattern (LBP) image
lbp-3D-m2_glcm	24	Gray-Level Co-occurrence Matrix (GLCM) extracted from lbp-3D-m2
lbp-3D-m1_glcm	24	Gray-Level Co-occurrence Matrix (GLCM) extracted from lbp-3D-m1
lbp-2D_glcm	24	Gray-Level Co-occurrence Matrix (GLCM) extracted from lbp-2D
gradient_glcm	24	Gray-Level Co-occurrence Matrix (GLCM) extracted from Image Gradient
logarithm_glcm	24	Gray-Level Co-occurrence Matrix (GLCM) extracted from logarithm
wavelet-HLH_glcm	24	Gray-Level Co-occurrence Matrix (GLCM) extracted from Wavelet (HLH) filtered image
wavelet-HHL_glcm	24	Gray-Level Co-occurrence Matrix (GLCM) extracted from wavelet-HHL
wavelet-LHH_glcm	24	Gray-Level Co-occurrence Matrix (GLCM) extracted from wavelet-LHH
wavelet-LLH_glcm	24	Gray-Level Co-occurrence Matrix (GLCM) extracted from wavelet-LLH
wavelet-LHL_glcm	24	Gray-Level Co-occurrence Matrix (GLCM) extracted from wavelet-LHL
squareroot_glcm	24	Gray-Level Co-occurrence Matrix (GLCM) extracted from squareroot
square_glcm	24	Gray-Level Co-occurrence Matrix (GLCM) extracted from Squared Image
wavelet-HLL_glcm	24	Gray-Level Co-occurrence Matrix (GLCM) extracted from Wavelet (HLL) filtered image
wavelet-HHH_glcm	24	Gray-Level Co-occurrence Matrix (GLCM) extracted from Wavelet (HHH) filtered image
wavelet-LLL_glcm	24	Gray-Level Co-occurrence Matrix (GLCM) extracted from Wavelet (LLL) filtered image
squareroot_firstorder	18	First-order statistics extracted from squareroot
wavelet-LHH_firstorder	18	First-order statistics extracted from wavelet-LHH
wavelet-HHL_firstorder	18	First-order statistics extracted from wavelet-HHL
wavelet-LHL_firstorder	18	First-order statistics extracted from wavelet-LHL
wavelet-LLH_firstorder	18	First-order statistics extracted from wavelet-LLH
lbp-3D-k_firstorder	18	First-order statistics extracted from 3D Local Binary Pattern (LBP) image
lbp-3D-m1_firstorder	18	First-order statistics extracted from lbp-3D-m1
lbp-2D_firstorder	18	First-order statistics extracted from lbp-2D
original_firstorder	18	First-order statistics extracted from Original Image
wavelet-HHH_firstorder	18	First-order statistics extracted from Wavelet (HHH) filtered image
wavelet-LLL_firstorder	18	First-order statistics extracted from Wavelet (LLL) filtered image
wavelet-HLH_firstorder	18	First-order statistics extracted from Wavelet (HLH) filtered image
wavelet-HLL_firstorder	18	First-order statistics extracted from Wavelet (HLL) filtered image
square_firstorder	18	First-order statistics extracted from Squared Image
logarithm_firstorder	18	First-order statistics extracted from logarithm
gradient_firstorder	18	First-order statistics extracted from Image Gradient
lbp-3D-m2_firstorder	18	First-order statistics extracted from lbp-3D-m2
exponential_firstorder	18	First-order statistics extracted from exponential
lbp-3D-k_glszm	16	Gray-Level Size Zone Matrix (GLSZM) extracted from 3D Local Binary Pattern (LBP) image
lbp-3D-m2_glszm	16	Gray-Level Size Zone Matrix (GLSZM) extracted from lbp-3D-m2

lbp-3D-k_glrlm	16	Gray-Level Run Length Matrix (GLRLM) extracted from 3D Local Binary Pattern (LBP) image
original_glrlm	16	Gray-Level Run Length Matrix (GLRLM) extracted from Original Image
exponential_glrlm	16	Gray-Level Run Length Matrix (GLRLM) extracted from exponential
original_glszm	16	Gray-Level Size Zone Matrix (GLSZM) extracted from Original Image
lbp-3D-m2_glrlm	16	Gray-Level Run Length Matrix (GLRLM) extracted from lbp-3D-m2
lbp-3D-m1_glszm	16	Gray-Level Size Zone Matrix (GLSZM) extracted from lbp-3D-m1
lbp-2D_glrlm	16	Gray-Level Run Length Matrix (GLRLM) extracted from lbp-2D
gradient_glszm	16	Gray-Level Size Zone Matrix (GLSZM) extracted from Image Gradient
exponential_glszm	16	Gray-Level Size Zone Matrix (GLSZM) extracted from exponential
wavelet-LLL_glszm	16	Gray-Level Size Zone Matrix (GLSZM) extracted from Wavelet (LLL) filtered image
wavelet-LLL_glrlm	16	Gray-Level Run Length Matrix (GLRLM) extracted from Wavelet (LLL) filtered image
wavelet-HHL_glrlm	16	Gray-Level Run Length Matrix (GLRLM) extracted from wavelet-HHL
wavelet-HHL_glszm	16	Gray-Level Size Zone Matrix (GLSZM) extracted from wavelet-HHL
wavelet-HHH_glszm	16	Gray-Level Size Zone Matrix (GLSZM) extracted from Wavelet (HHH) filtered image
wavelet-HLH_glrlm	16	Gray-Level Run Length Matrix (GLRLM) extracted from Wavelet (HLH) filtered image
wavelet-LHH_glszm	16	Gray-Level Size Zone Matrix (GLSZM) extracted from wavelet-LHH
wavelet-HLL_glrlm	16	Gray-Level Run Length Matrix (GLRLM) extracted from Wavelet (HLL) filtered image
wavelet-HLH_glszm	16	Gray-Level Size Zone Matrix (GLSZM) extracted from Wavelet (HLH) filtered image
wavelet-HHH_glrlm	16	Gray-Level Run Length Matrix (GLRLM) extracted from Wavelet (HHH) filtered image
squareroot_glszm	16	Gray-Level Size Zone Matrix (GLSZM) extracted from squareroot
squareroot_glrlm	16	Gray-Level Run Length Matrix (GLRLM) extracted from squareroot
square_glrlm	16	Gray-Level Run Length Matrix (GLRLM) extracted from Squared Image
square_glszm	16	Gray-Level Size Zone Matrix (GLSZM) extracted from Squared Image
logarithm_glrlm	16	Gray-Level Run Length Matrix (GLRLM) extracted from logarithm
logarithm_glszm	16	Gray-Level Size Zone Matrix (GLSZM) extracted from logarithm
wavelet-LHH_glrlm	16	Gray-Level Run Length Matrix (GLRLM) extracted from wavelet-LHH
gradient_glrlm	16	Gray-Level Run Length Matrix (GLRLM) extracted from Image Gradient
lbp-2D_glszm	16	Gray-Level Size Zone Matrix (GLSZM) extracted from lbp-2D
lbp-3D-m1_glrlm	16	Gray-Level Run Length Matrix (GLRLM) extracted from lbp-3D-m1
wavelet-LLH_glszm	16	Gray-Level Size Zone Matrix (GLSZM) extracted from wavelet-LLH
wavelet-HLL_glszm	16	Gray-Level Size Zone Matrix (GLSZM) extracted from Wavelet (HLL) filtered image
wavelet-LHL_glrlm	16	Gray-Level Run Length Matrix (GLRLM) extracted from wavelet-LHL
wavelet-LLH_glrlm	16	Gray-Level Run Length Matrix (GLRLM) extracted from wavelet-LLH
wavelet-LHL_glszm	16	Gray-Level Size Zone Matrix (GLSZM) extracted from wavelet-LHL
exponential_gldm	14	Gray-Level Dependence Matrix (GLDM) extracted from exponential
original_gldm	14	Gray-Level Dependence Matrix (GLDM) extracted from Original Image
original_shape	14	shape extracted from Original Image
lbp-3D-m1_gldm	14	Gray-Level Dependence Matrix (GLDM) extracted from lbp-3D-m1
lbp-2D_gldm	14	Gray-Level Dependence Matrix (GLDM) extracted from lbp-2D
lbp-3D-m2_gldm	14	Gray-Level Dependence Matrix (GLDM) extracted from lbp-3D-m2
lbp-3D-k_gldm	14	Gray-Level Dependence Matrix (GLDM) extracted from 3D Local Binary Pattern (LBP) image
wavelet-LLL_gldm	14	Gray-Level Dependence Matrix (GLDM) extracted from Wavelet (LLL) filtered image
wavelet-HHH_gldm	14	Gray-Level Dependence Matrix (GLDM) extracted from Wavelet (HHH) filtered image

wavelet-HHL_gldm	14	Gray-Level Dependence Matrix (GLDM) extracted from wavelet-HHL
wavelet-HLH_gldm	14	Gray-Level Dependence Matrix (GLDM) extracted from Wavelet (HLH) filtered image
wavelet-HLL_gldm	14	Gray-Level Dependence Matrix (GLDM) extracted from Wavelet (HLL) filtered image
wavelet-LLH_gldm	14	Gray-Level Dependence Matrix (GLDM) extracted from wavelet-LLH
wavelet-LHL_gldm	14	Gray-Level Dependence Matrix (GLDM) extracted from wavelet-LHL
wavelet-LHH_gldm	14	Gray-Level Dependence Matrix (GLDM) extracted from wavelet-LHH
squareroot_gldm	14	Gray-Level Dependence Matrix (GLDM) extracted from squareroot
gradient_gldm	14	Gray-Level Dependence Matrix (GLDM) extracted from Image Gradient
logarithm_gldm	14	Gray-Level Dependence Matrix (GLDM) extracted from logarithm
square_gldm	14	Gray-Level Dependence Matrix (GLDM) extracted from Squared Image
original_ngtdm	5	Neighborhood Gray-Tone Difference Matrix (NGTDM) extracted from Original Image
exponential_ngtdm	5	Neighborhood Gray-Tone Difference Matrix (NGTDM) extracted from exponential
lbp-3D-m1_ngtdm	5	Neighborhood Gray-Tone Difference Matrix (NGTDM) extracted from lbp-3D-m1
lbp-2D_ngtdm	5	Neighborhood Gray-Tone Difference Matrix (NGTDM) extracted from lbp-2D
wavelet-LHL_ngtdm	5	Neighborhood Gray-Tone Difference Matrix (NGTDM) extracted from wavelet-LHL
wavelet-LLH_ngtdm	5	Neighborhood Gray-Tone Difference Matrix (NGTDM) extracted from wavelet-LLH
lbp-3D-k_ngtdm	5	Neighborhood Gray-Tone Difference Matrix (NGTDM) extracted from 3D Local Binary Pattern (LBP) image
lbp-3D-m2_ngtdm	5	Neighborhood Gray-Tone Difference Matrix (NGTDM) extracted from lbp-3D-m2
gradient_ngtdm	5	Neighborhood Gray-Tone Difference Matrix (NGTDM) extracted from Image Gradient
logarithm_ngtdm	5	Neighborhood Gray-Tone Difference Matrix (NGTDM) extracted from logarithm
square_ngtdm	5	Neighborhood Gray-Tone Difference Matrix (NGTDM) extracted from Squared Image
squareroot_ngtdm	5	Neighborhood Gray-Tone Difference Matrix (NGTDM) extracted from squareroot
wavelet-HLH_ngtdm	5	Neighborhood Gray-Tone Difference Matrix (NGTDM) extracted from Wavelet (HLH) filtered image
wavelet-HHL_ngtdm	5	Neighborhood Gray-Tone Difference Matrix (NGTDM) extracted from wavelet-HHL
wavelet-LHH_ngtdm	5	Neighborhood Gray-Tone Difference Matrix (NGTDM) extracted from wavelet-LHH
wavelet-HLL_ngtdm	5	Neighborhood Gray-Tone Difference Matrix (NGTDM) extracted from Wavelet (HLL) filtered image
wavelet-HHH_ngtdm	5	Neighborhood Gray-Tone Difference Matrix (NGTDM) extracted from Wavelet (HHH) filtered image
wavelet-LLL_ngtdm	5	Neighborhood Gray-Tone Difference Matrix (NGTDM) extracted from Wavelet (LLL) filtered image
diagnostics_Image-original	3	Image-original features
diagnostics_Mask-original	2	Mask-original features
